# Encoded-Enhancement of THz Metasurface Figure of Merit for Label-Free Sensing

**DOI:** 10.3390/s19112544

**Published:** 2019-06-04

**Authors:** Gian Paolo Papari, Can Koral, Antonello Andreone

**Affiliations:** 1 Department of Physics, University of Naples “Federico II”, and CNR-SPIN, I-80125 Naples, Italy; andreone@unina.it; 2 Istituto Nazionale di Fisica Nucleare (INFN), Naples Unit, Complesso Universitario di Monte S. Angelo, via Cinthia, I-80126 Naples, Italy; ckoral@na.infn.it

**Keywords:** terahertz metasurface, label-free sensing, quality factor

## Abstract

We describe an experimental strategy for the use of Terahertz (THz) metasurfaces as a platform for label-free wide range detection of the dielectric function in biological fluids. Specifically, we propose a metagrid (MG), opportunely infiltrated with a fluid and then capped, as the reference structure for sensing experiments with a high reproducibility character. By combining experiments and full-wave simulations of the transmission *T* of such a structure, we introduce a reliable set up where the volume of the involved analyte in each unit cell is precisely determined. The unavoidable decrease in the quality factor of the intrinsic resonances due to the lossy fluid and cap layer is circumvented using an appropriate transformation of *T* that amplifies the change in the MG intrinsic resonances, improving in such a way the sensor sensitivity to values close to the experimental limits. The transformed signal features delta-like peaks enabling an easy readout of frequency positions at resonances.

## 1. Introduction

Metasurfaces (MS) are artificial 2D-structures typically realized by patterning a metallic layer in an array of resonators distributed over a dielectric substrate [1,2,3,4,5]. Depending on the geometrical shape of each single resonator, on the unit cell periodicity, and on the substrate dielectric properties, peculiar resonating features can be “engineered” in the transmission/reflection characteristics of a MS. The frequency position of each resonance $f_{0}∼1/\sqrt{L_{0}C_{0}}$, where $C_{0}$ and $L_{0}$ describe the effective capacitive and inductive coupling respectively between the impinging radiation and the metallo-dielectric structures, is potentially sensitive to any change of the electromagnetic environment [6,7,8]. This effect is stimulating an extensive application of MS in the THz band, where sensing experiments enjoy a series of advantages with respect to other portions of the electromagnetic spectrum. In this frequency region in fact the geometrical features needed for the MS design are easily achieved by standard UV lithography, in contrast with optical/infrared band operating structures, where nanoscale fabrication demands more working time and higher costs since they are based on electron/ion lithography (EBL/FIB) [9,10,11]. Moreover, in the THz region it is quite easy to gather comprehensive information since measurements are based on the coherent time domain approach and therefore allow direct access to the complex dielectric response of the material under test. However, a typical retrieval process [12,13] is based on the computation of curves (arrays) made of hundreds of points. Instead using a metasurface, computational efforts are drastically reduced since in this case the sensing experiment is based on the frequency shift induced in just a few resonances [14]. 

Previous research strategies focused on the test of a dropped solution after its complete evaporation over the MS, to measure the transmission *T* change due, for instance, to the deposition of proteins [10,15,16], bacteria [17] or antibiotics [18]. Although this technique allows the detection of single cells deposited on the unit cell, it makes it very difficult to quantify the analyte dielectric function since it lacks information on its effective mass. Sometimes, however, the control on the sample volume is mandatory in order to use a mean field approach [19,20], for instance to extract information on analytes composed of suspended particles, or simply to numerically simulate a reproducible experiment.

A handy and suitable way to manage the analyte volume is to use MS in the shape of metagrids consisting of an array of periodic cells designed to present holes [21]. By capping the structure with an appropriate layer (capped metagrid, CMG), the analyte remains trapped in the specific volume of each unit cell. Differently from sensing experiments employing cuvettes [22], this structure behaves as a resonating cavity that can map with high accuracy the analyte. Furthermore, metagrids can be opportunely designed to feature in a simple way a series of resonances that can be used as markers for mapping the analyte dielectric constant over a wide band, upper bounded by the diffraction cutoff frequency *f_p_* = *c*/*p* [21]. 

The sensitivity S of a metagrid (MG) is then directly related to the shift in resonance peaks (measured in wavelength $\Delta \lambda_{0}$ [23]) as a function of the analyte refractive index $n_{x}$:$S=\Delta \lambda_{0}/\Delta n_{x}$. A resonance having a high-quality factor certainly improves the spectroscopic signature and sensitivity because it enhances the resolution and allows an easy and fast acquisition of the measurement. On the other hand, the presence of a cap layer unavoidably lowers the resonance quality factor of the bare MG, and the sensitivity of the CMG is degraded as well because the accuracy of *S* depends on the width of each resonance [24]. The low quality factor of surface plasmon polariton (SPP) resonances is a widespread issue [25,26,27] in spectroscopy, and alternative solutions to improve the quality factor of resonances are in demand.

In this paper, we provide an algorithm that recognizes the “local” quality factor of a generic *T* curve, enhancing the mapping of each resonance peak $f_{0}\left(\varepsilon_{x}\right)$ versus the dielectric constant of the analyte $\varepsilon_{x}=n_{x}^{2}$. We apply this procedure to the experimental THz signal transmitted through a CMG obtained capping an empty grid-shaped copper metasurface realized using a standard printed circuit board (PCB) technology with a glass cover slab. We then simulate the response for $\varepsilon_{x}$ ranging between 1 and 20 to mimic the dielectric function of an analyte based on a biological fluid [22,28,29,30]. 

## 2. Materials and Methods

A THz metasurface was fabricated on a PCB (FR4) having a thickness d_1_ = 160 μm. The copper layer was patterned in shape of a square grid presenting a periodicity *p* = 600 μm. The vertical and horizontal wires 300 μm wide composed a grid having thickness d_2_ = 30 μm. The cap layer consisted of a glass slab with thickness d_3_ = 150 μm.

**Figure 1:**
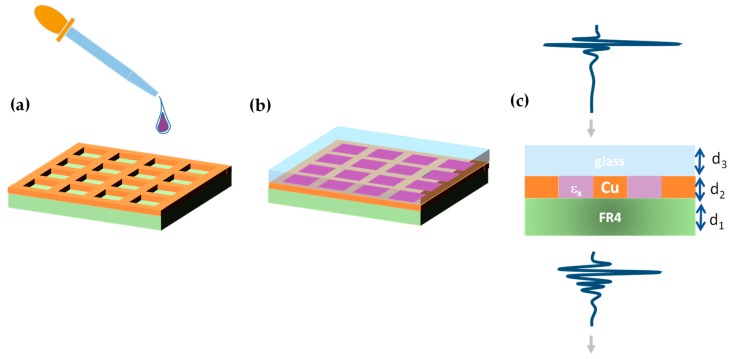
(**a**,**b**): sketch of the steps for the preparation of the capped metagrid (CMG) sample with analyte. (**c**): Measurement setup configuration and section view of the capped metasurface, where d_1_ = 160 μm, d_2_ = 30 μm, d_3_ = 150 μm.

The steps to prepare the CMG for measurements are sketched in Figure 1a,b. First, a liquid analyte was poured on the MS. Subsequently, the cover was placed and pressed in order to obtain the maximum adherence between the two surfaces. This ensures that liquid in excess dropped out of the structure to guarantee that the analyte thickness approximately coincided with d_2_.

The proposed sensor for biological fluids is very simple to realize and—as long as the analyte does not contaminate the MS—is reusable improving the cost-effectiveness of the device.

THz time domain spectroscopy (TDS) was performed employing a Menlo^®^ optical apparatus. The system technology is based on photoconductive antennas which are excited by a pulsed laser at wavelength λ = 1550 nm. The THz signal was collimated by using TPX (Polymethylpentene) lenses. This resulted in a beam having plane wave-like characteristics, very close to the simulated configuration. To remove any detrimental absorption due to water vapor, measurements were performed in a purging box keeping the level of humidity lower than 0.1%. In order to perform experiments keeping the CMG parallel to the optical board and avoid the analyte leakage, the THz pulse was transmitted vertically. A sketch of the measurement setup is presented in Figure 1c, where all relevant quantities are shown.

## 3. Results

**Figure 2:**
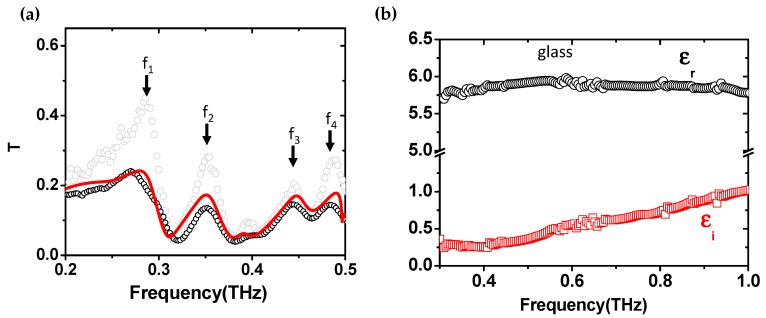
(**a**) Grey and black circles represent the experimental Terahertz (THz) signal transmission of the bare and capped (without analyte) metagrid respectively. The frequencies *f*_1–4_ indicate the most sensitive transmission resonances. The red curve is the full wave simulation of the CMG transmission. (**b**) Values of the real (black circles) and imaginary (red squares) part of the dielectric function for the cover glass slab vs. frequency up to 1 THz.

A detailed analysis of the modes related to the enhanced transmission mechanisms responsible for the peaks in *T* has been reported in [21]. The interest in the analysis of the CMG spectra lies in the frequency band *f* < *f_p_*, where the grid transmission response is dominated by collective modes and diffraction losses are low [21]. In this region four resonances can be pointed out, *f*_1_ = 0.28 THz, *f*_2_ = 0.35 THz, *f*_3_ = 0.45 THz, *f*_4_ = 0.48 THz, which potentially represent the most sensitive features in the *T* spectrum. In Figure 2a the measured transmitted signal *T* of the empty CMG is reported as black dots, and compared with a full wave electromagnetic simulation (continuous red curve) performed using CST Microwave Studio^®^. The measured *T* of the uncapped MG is reported as well as grey dots, showing that the overlapping of the glass cover did not significantly change the frequency position of the main resonances. This is because the dependence of Bragg modes is mostly on the grid periodicity and on the dielectric constant of the FR4 where the metallic layer is deposited [31]. Simulation of the bare metagrid basically followed the experimental curve [20], and it is not reported here.

The thin glass slab was characterized stand-alone and its complex dielectric function $\tilde{\varepsilon }=\varepsilon_{r}+i\varepsilon_{i}$ retrieved, as reported in Figure 2b. While the real part $\varepsilon_{r}$ stayed approximately constant in the band under investigation, the imaginary part $\varepsilon_{i}$ started increasing at around 0.5 THz, indicating that glass absorbs most of the signal above that frequency, which sets an upper limit in its use as cap layer.

The presence of a cap layer along with the insertion of a dissipative analyte always degrades the resonance quality, in terms of both amplitude and width, sometimes in an unpredictable manner. 

The quality factor Q = *f*_0_/Δ*f*_0_(−3dB) [32] of the selected resonances decorating the *T* of the bare metagrid ranged between 10 and 20, decreasing on average by 30% capping the metasurface. As a result, dephasing time—simply defined in THz spectroscopy as *τ* = 1/Δ*f*_0_(−3dB) [33,34]—decreased too, reducing to values close to 15 ps, still among the highest ones available in literature [34].

The reduction in the figure of merit and therefore in *τ* can be circumvented by introducing an algorithm that magnifies the changes in the signal transmission produced by the analyte, independently of the effective quality factor of a resonance placed at *f*_0_. 

Mapping the *T* values as a function of frequency and analyte dielectric constant, we can evaluate a function dimensionally given by a frequency width ${ℱ}_{T}^{\left(n\right)}\;\left(f,\varepsilon_{x}\right)$ underneath each point of the signal transmission and retrieve in this way a contour plot $Q_{T}^{\left(n\right)}\;\left(f,\varepsilon_{x}\right)$, simply obtained by dividing ${ℱ}_{T}^{\left(n\right)}$ for the frequency bin δf:

(1) $$Q_{T_{i}}^{\left(n\right)}=\frac{{ℱ}_{T_{i}}^{\left(n\right)}}{\delta f}=\frac{1}{T_{max,i}-T_{min,i}}\left[\left(\sum_{j=-n}^{n}T_{i+j}\right)-2n\cdot T_{min,i}\right]=2n\frac{T_{avg,i}^{\left(n\right)}-T_{min,i}}{T_{max,i}-T_{min,i}},$$

where *i* indicates the *i*-th data point, *n* is an integer defining the semi-interval over which the local ${ℱ}_{T}^{\left(n\right)}$ is calculated, $T_{max/min,i}$ represent the maximum/minimum transmission values within the interval $\left\{T_{i+n},T_{i-n}\right\}$, and $T_{avg,i}^{\left(n\right)}$ is the average transmission in the same interval. 

**Figure 3:**
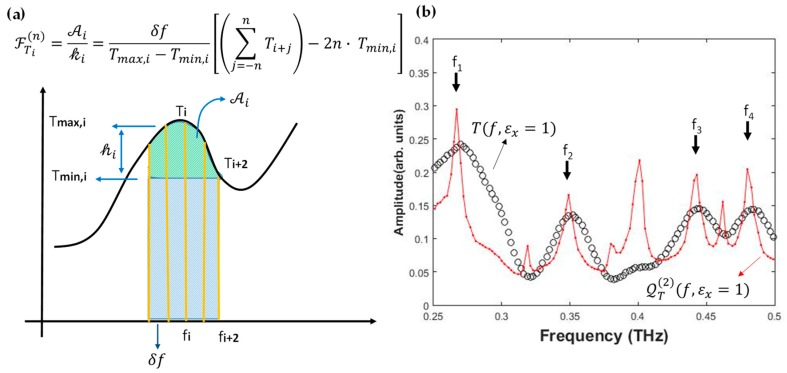
(**a**) Graphical representation of the function ${ℱ}_{T_{i}}^{\left(2\right)}$. The square bracket in the formula is proportional to the green area underneath the transmission point *T_i_*. The frequency interval is straightforwardly achieved by dividing the green area by the height *T_max,i_* − *T_min,i_* relative to the points ensemble $\left\{T_{i-n}-T_{i+n}\right\}$. The “local” quality factor $Q_{T}^{\left(2\right)}$ is then obtained by dividing ${ℱ}_{T_{i}}^{\left(2\right)}$ for the frequency bin *δf*. (**b**) Comparison between the measured transmission of the empty $\left(\varepsilon_{x}=1\right)$ CMG (black open circles) and $Q_{T}^{\left(2\right)}$ (scaled for the sake of clarity).

In Figure 3a, a sketch explaining the conceptual meaning of ${ℱ}_{T}^{\left(n\right)}$ for *n* = 2 is reported. One can easily see that the quantity in the square brackets of Equation (1) identifies the green dashed area in the figure, so that its ratio with $T_{max,i}-T_{min,i}$ provides a frequency interval not to be mistaken for the resonance width. In this respect $Q_{T_{i}}^{\left(n\right)}$ represents a gauge of the growth rate of the transmitted signal *T*(*f*) “area” and quantifies therefore a “local” figure of merit. It is worth noting that, in spite of the fact that $Q_{T}^{\left(n\right)}$ resembles a differential quantity, it is not proportional to the simple *T*-derivative that actually yields zero in correspondence of extreme points in transmission. 

The last expression in Equation (1) highlights that $Q_{T}^{\left(n\right)}$ is different from zero only when $T_{avg,i}^{\left(n\right)}-T_{min,i}\neq 0$, which allows the mapping of all extreme points of *T* in “spikes” having width of the order of $\Delta f_{0}∼2n\cdot \delta f$. This highlights the main role of $Q_{T}^{\left(n\right)}$ that, independently of the intrinsic figure of merit for the selected metagrid modes, maps them in resonances which are directly close to the experimental limit achieved for *n* = 1, $\Delta f_{0}^{min}=2\cdot \delta f$. In our case *δf* = 5 GHz was given by the experimental sampling interval in the frequency domain, providing therefore maxima in the local quality factor between 20 and 40 in the range 0.2–0.4 THz.

With regard to *n*, small values tend to produce artifacts because of noise, whereas values too high average out the resonance peaks. In applying the algorithm in Equation (1) to the experimental *T*, we observed that the choice *n* = 2 was a good compromise, although up to *n* = 4 peaks in $Q_{T}^{\left(n\right)}$ were still clearly observable. The function $Q_{T}^{\left(n\right)}\left(f\right)$ for *ε_x_* = 1 is reported in Figure 3b as a dash-dotted red line, displaying as expected very sharp peaks in correspondence of the transmission resonances. This translated to a threefold improvement for each mode quality factor, increasing on average from a value ~10 to ~30. More importantly, the striking advantage here was to deal with delta-like features allowing an easy readout and a computationally fast access to the calibration curves $f_{0}\left(\varepsilon_{x}\right)$.

## 4. Discussion

**Figure 4:**
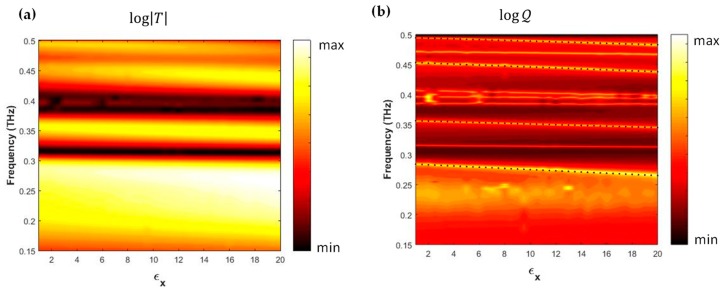
(**a**) False color map (log scale) of the transmission *T*(*f*, *ε_x_*) for the capped metagrid. (**b**) Same for $Q_{T}^{\left(2\right)}\left(f,\varepsilon_{x}\right)$. Dotted lines highlight the behavior of $f_{1–4}\left(\varepsilon_{x}\right)$, selected as the most sensitive modes in the CMG structure.

In Figure 4, the core result of the paper is reported presenting for the capped metagrid contour plots—expressed in dB—*T*(*f*, *ε_x_*) and $Q_{T}^{\left(2\right)}$ in panels (a) and (b) respectively. As already observed in Figure 3b, it is interesting to note how the algorithm in Equation (1) changed the quality of information on resonance dynamics passing from *T*(*f*, *ε_x_*) to $Q_{T}^{\left(2\right)}\left(f,\varepsilon_{x}\right)$. 

In this way the four selected resonances *f*_1–4_ showed wider dynamics as a function of $\varepsilon_{x}$, as plotted in Figure 4b, allowing the definition of four different sensing bands given by the single frequency shifts $\Delta f_{i}=f_{i}\left(\varepsilon_{x}=20\right)-f_{i}\left(\varepsilon_{x}=1\right)∼0.02\;\mathrm{THz}$. Within an error of a few percent each resonance frequency can be simply written:

(2) $$f_{i}\left(\varepsilon_{x}\right)=\left(a_{i}\varepsilon_{x}+b_{i}\right)\;\mathrm{THz}$$

where the parameters $\left(a_{i},b_{i}\right),$ with *i* = 1–4, are listed in Table 1. Each calibration curve is reported in Figure 4b as a dotted line. The feeble peak in *T* at about 0.4 THz has been neglected because its experimental observation is too hard to follow in the presence of whatever small losses were introduced by a real analyte.

In principle, using the algorithm given by Equation (1), one can also track frequency changes relatively to a minimum in the signal transmission. However, this is usually not a “smart” marker for sensing because it can be easily misplaced if close to the noise level, introducing therefore a relevant uncertainty in the calibration curve. Furthermore, in our case minima in the signal resonances (respectively at 0.33, 0.47, 0.38, 0.42 THz) were much less sensitive to any variation of $\varepsilon_{x}$ because the corresponding electric field mostly oscillated at the metal-dielectric boundary. In contrast, in a mode maximum, the electric field (as displacement current [14]) builds up in the metallic hole volume [21], where the analyte was stored. 

**Figure 5:**
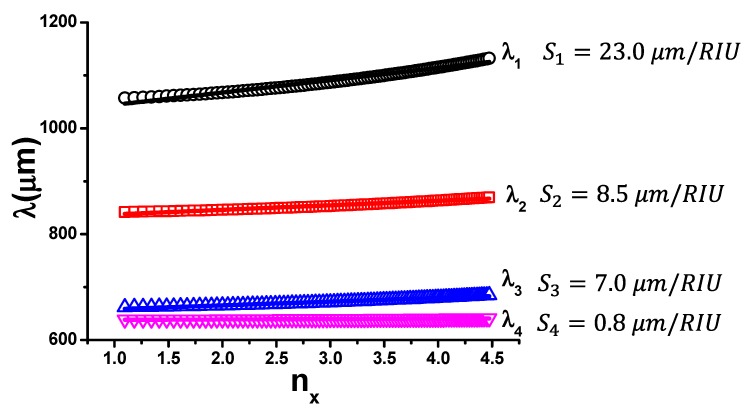
Data points display the resonance frequencies vs, $n_{x}$ retrieved from Figure 4b expressed in terms of wavelength *λ_i_* = *c*/*f_i_*. Full lines represent the linear fits providing the sensitivity values $S_{i}$ for each curve.

In Figure 5, we report as dotted curves the linear calibration curves given in Equation (2) and expressed in terms of wavelength $(\lambda_{i}=c/f_{i}$) versus refractive index $n_{x}=\sqrt{\varepsilon_{x}}$. A linear fit of the four curves implies an error that in the worst case (*f*_1_) is smaller than 3%. The angular coefficient of each linear fit provides the sensitivity $S_{i}=d\lambda_{i}/dn_{x}$ related to each resonance. Estimated values are close to the highest presently recorded in literature [6,35,36] and are reported next to each $\lambda_{i}\left(n_{x}\right)$ curve. We argue that the decrease in sensitivity as a function of the resonance order is possibly due to the mode transition from a dipolar (*f*_1_) to an hexapolar (*f*_4_) character, where the electric field tends to concentrate within the substrate [21], weakening its dependence on analyte $n_{x}$ change.

Further improvement is certainly possible increasing the overall transparency of the CMG structure, by resorting to materials with reduced losses for both the substrate and the cap layer.

## 5. Conclusions

We have explored the sensing properties of a capped grid metasurface operating in the THz region when infiltrated with a liquid. Using a combination of measurements and full-wave simulations, we have analyzed the potential sensitivity of a specific sensor realized employing a copper metagrid, routinely patterned on a PCB and capped with a thin layer of glass. The frequency shifts in the CMG transmission resonances due to the change of analyte dielectric constant $\varepsilon_{x}$ in the range 1–20 have been simulated. The full control of the analyte volume inside each unit cell confers a high reproducibility on future experiments. The unavoidable degradation of the MS intrinsic features due to the presence of a lossy liquid is healed resorting to a transformation that maps the measured transmission *T* in terms of a local figure of merit $Q_{T}^{\left(n\right)}$, that allows at the same time a magnified effect on a resonance placed at $f_{0}$ and an easy readout of the calibration curves $f_{0}\left(\varepsilon_{x}\right)$.

The best advantage in the introduction of the parameter $Q_{T}^{\left(n\right)}$ is to provide a consistent enhancement of the measure sensitivity and robustness, independently from the quality factor of the employed MS and regardless of the external conditions (mostly, the losses in the liquid under test). $Q_{T}^{\left(n\right)}$ is shaped up so that it allows (by varying *n*) the best *T* transformation to be found and therefore optimize the figure of merit achievable from the experimental session. This procedure provides an alternative and—we believe—more efficient strategy, based on a simple use of data processing, to deal with and enrich signal quality. 

In the CMG under test, four different transmission peaks allow the dielectric constant of the analyte to be sensed almost over the entire operational band 0.3–0.5 THz, with very high estimated sensitivities, close to record values reported in literature. This suggests that THz capped metagrids may be profitably used as reference platforms for accurate and fast label free sensing of biological fluids. It can be used, as an example, to monitor either the growth of cancer cells [37] or their degree of damage induced by ionizing radiation [38]. Furthermore, the algorithm proposed in Equation (1) is particularly suitable for further improvements accounting in an easy way for the signal derivative too, for instance to select/display only the maxima (or minima) in the transmission.

## Figures and Tables

**Table 1:** Fitting parameters used in Equation (2) according to the resonance index *i*. DCU stands for dielectric constant unit.

*i*	a_i_ (× 10^−4^) [THz/DCU]	b_i_ (× 10^−4^) [THz]
1	−1	2850
2	−6	3560
3	−8	4530
4	−6	4940
